# Accrual and retention of diverse patients in psychosocial cancer clinical trials

**DOI:** 10.1017/cts.2022.380

**Published:** 2022-04-01

**Authors:** Grace Ann Hanvey, Adaixa Padron, Elizabeth L. Kacel, Gabriel Cartagena, Kelsey C. Bacharz, Christina S. McCrae, Michael E. Robinson, Lori B. Waxenberg, Michael H. Antoni, Richard B. Berry, Gregory S. Schultz, Jacqueline Castagno, Deidre B. Pereira

**Affiliations:** 1 Department of Clinical and Health Psychology, University of Florida, Gainesville, FL, USA; 2 Department of Psychology, University of Miami, Coral Gables, FL, USA; 3 Division of Pulmonary, Critical Care, and Sleep Medicine, University of Florida, Gainesville, FL, USA; 4 Department of Obstetrics and Gynecology, University of Florida, Gainesville, FL, USA

**Keywords:** Cancer, clinical trials, recruitment, retention, health disparities

## Abstract

**Background:**

Minority and older adult patients remain underrepresented in cancer clinical trials (CCTs). The current study sought to examine sociodemographic inequities in CCT interest, eligibility, enrollment, decline motivation, and attrition across two psychosocial CCTs for gynecologic, gastrointestinal, and thoracic cancers.

**Methods:**

Patients were approached for recruitment to one of two interventions: (1) a randomized control trial (RCT) examining effects of a cognitive-behavioral intervention targeting sleep, pain, mood, cytokines, and cortisol following surgery, or (2) a yoga intervention to determine its feasibility, acceptability, and effects on mitigating distress. Prospective RCT participants were queried about interest and screened for eligibility. All eligible patients across trials were offered enrollment. Patients who declined yoga intervention enrollment provided reasons for decline. Sociodemographic predictors of enrollment decisions and attrition were explored.

**Results:**

No sociodemographic differences in RCT interest were observed, and older patients were more likely to be ineligible. Eligible Hispanic patients across trials were significantly more likely to enroll than non-Hispanic patients. Sociodemographic factors predicted differences in decline motivation. In one trial, individuals originating from more urban areas were more likely to prematurely discontinue participation.

**Discussion:**

These results corroborate evidence of no significant differences in CCT interest across minority groups, with older adults less likely to fulfill eligibility criteria. While absolute Hispanic enrollment was modest, Hispanic patients were more likely to enroll relative to non-Hispanic patients. Additional sociodemographic trends were noted in decline motivation and geographical prediction of attrition. Further investigation is necessary to better understand inequities, barriers, and best recruitment practices for representative CCTs.

## Introduction

Despite the necessity of representative cancer clinical trials (CCTs) in effective disease treatment, CCT participation is significantly limited across the general cancer population. Early investigations of CCT participation trends reveal that less than 5% of cancer patients enroll in such trials [1] with minimal improvement demonstrated in more recent reports [2,3], reflecting urgency to promote improvements in accrual and attrition rates [1]. Evidence illustrates that CCT recruitment outcomes have been especially poor among older adults and racial and/or ethnic minority patients [1,3-5]. These disparities have been attributed to the disproportionate impact of certain barriers on these vulnerable populations, including socioeconomic status (SES) [2,6]; recruitment and trial locations [7,8]; health literacy [9]; limited cultural sensitivity [5,7]; eligibility criteria [2,6]; clinical infrastructure [7,10]; researcher and provider bias [2,3,6,11]; and inequalities in awareness [6,7]. Further, evidence indicates that little progress has been made in attenuating these disparities over time [12]. Notwithstanding these inequalities, CCT participation interest is largely independent of sociodemographic factors [2,5]. Given elevated disease burden and poorer cancer outcomes among the underserved, it is critical that trial participation disparities are investigated [7,9]. Moreover, recent literature indicates that implementation of patient-centered, culturally sensitive recruitment strategies tailored toward CCT engagement among underrepresented populations in cancer research have yielded positive implications for attenuating inequities in CCT participation [13-15].

Available literature on representative CCT recruitment primarily investigates therapeutic, rather than behavioral, interventions [6,16]. Furthermore, reviews that explore accrual and retention do not always distinguish potential differences between therapeutic and behavioral CCTs [3,6]. Thus, little is known about how participation disparities may be unique for marginalized groups in behavioral CCTs. However, of the studies to date including a behavioral component, data suggest potentially fewer barriers to enrollment and better recruitment outcomes compared to tumor-directed trials [9,15,17].

The purpose of the present study was to examine relationships among sociodemographic characteristics, accrual, and attrition outcomes in two psychosocial CCTs. Aim 1 examined sociodemographic differences in study enrollment status among eligible patients, eligibility status among all approached patients, interest level in participation among nonenrollees, and reasons for study decline. It was hypothesized that older participants would be less likely to fulfill eligibility criteria than younger participants; eligible minority patients would be less likely to enroll than nonminority patients; and that there would be no differences in study interest by age, race, and/or ethnicity.

Aim 2 examined sociodemographic characteristics, in addition to rurality status, and baseline intervention targets as predictors of study attrition. It was hypothesized that participants who were older, minorities, of lower SES, residing in more rural locations, or with greater baseline depressive and anxious symptomatology, sleep disturbance, and pain would demonstrate higher attrition.

## Materials and Methods

### Study Outcomes and Data Sources

Two behavioral CCTs – Trial 1 a cognitive-behavioral, randomized clinical trial intervention for insomnia and pain (CBTi.p.) and Trial 2 a single-arm, pilot yoga intervention – provided data for the present study. The trial(s) utilized for each set of analyses were contingent upon available data (Table 1).

**Table 1.** Study aims, outcomes, and data sources

Aim number	Outcome number	Trial 1 (CBT Trial) individually	Trial 2 (Yoga Trial) individually	Trials 1 and 2 combined
Aim 1	Outcome 1: Enrollment status among eligible patients			X
	Outcome 2: Eligibility status among all approached patients	X		
	Outcome 3: Level of interest in study participation among nonenrollees	X		
	Outcome 4: Trends in reasons for study decline		X	
Aim 2	Outcome 5: Study attrition	X	X	

CBT, cognitive behavioral therapy.

Four study outcomes were examined for Aim 1. Enrollment status among eligible patients (Outcome 1) was assessed utilizing data from both trials combined. Eligibility status among all approached patients (Outcome 2) and interest in study participation among nonenrollees (Outcome 3) were evaluated using data from the CBTi.p. intervention only, hereafter referred to as “Trial 1.” Trends in reasons for study decline (Outcome 4) were evaluated using data from the yoga intervention only, hereafter referred to as “Trial 2.”

Attrition for each study was examined for Aim 2. All study procedures for both interventions were approved by the UF Institutional Review Board (Trial 1: IRB201600679, Trial 2: IRB201700079). Procedures and measures are described below by trial, with the trials included in each set of analyses specified under “Statistical Analyses.”

### Trial 1: CBTi.p.

#### Participants, recruitment, and procedures

Individuals with suspected gynecologic cancers were approached for enrollment in a parallel, randomized clinical trial examining the effects of an individual CBTi.p. intervention enrolling from 2009 to 2017 to achieve adequate sample size (NCT02609880; https://clinicaltrials.gov/ct2/show/NCT02609880), funded by the National Cancer Institute. Doctoral students in clinical health psychology conducting research under the Principal Investigator’s (PI) Psycho-Oncology Laboratory engaged in daily health record reviews for patients scheduled for appointments at the UF Health Gynecologic Oncology Clinic to preliminarily screen for potentially eligible patients. Inclusion criteria were (1) ≥18 years of age, (2) scheduling for resection, debulking, or cytoreduction for incidence of confirmed or suspected gynecologic malignancies, and (3) English fluency. Exclusion criteria were (1) severe, uncontrolled psychopathology and/or suicidality, (2) Bipolar Disorder, (3) Neurocognitive Disorder, (4) seizure disorder, (5) current participation in another nonpharmacological sleep treatment, (6) sleep apnea or periodic limb movement disorder, and (7) estimated survival <6 months.

Prior to approval for enrolling patients, each doctoral student was trained by the PI in patient-centered approaches to facilitating screening, recruitment, and informed consent procedures, including training related to culturally competent screening, answering questions about the trial in the context of diverse psychosocial experiences, and addressing cultural health beliefs embodied within each approached patient. Prospective enrolling doctoral students were master’s-level therapists trained in psycho-oncological services with direct clinical foci in providing services to underserved populations in cancer care and integrated these experiences into the patient-centered, culturally sensitive screening and recruitment procedure paradigm. Following successful mock recruitment procedures with the PI, wherein diverse concerns were modeled and addressed, doctoral students were approved to enroll patients.

Approved enrolling students approached all preliminarily eligible patients and screened them for subjective sleep complaints (i.e., Sleep Quality Index >5 [18] or met Research Diagnostic Criteria “A” for Insomnia Disorder [19]). Prior to approaching each eligible patient, enrolling students consulted with the attending physician, discussing suitability for approach and study engagement as well as any psychosocial barriers the patient may experience to participation. Patients who were approved for approach, consented to eligibility screening, fulfilled study criteria, and were willing to be randomized were invited to complete written informed consent procedures. Individuals who declined participation were asked to identify their level of study interest and their decline reason(s). Additional measures were administered to exclude individuals with active suicidality and/or severe psychopathology [20,21]. Remaining eligible patients underwent psychosocial assessment, peripheral venous blood draw, and salivary cortisol collection.

Six to eight weeks following surgery, participants returned for postsurgical follow-up and repeated assessment. Eligibility criteria for randomization included (1) confirmed gynecologic cancer or borderline ovarian tumor, (2) presence of at least subclinical insomnia via 14 days of sleep diaries, and (3) discontinuation of prescribed sleep medications for ≥1 month or stabilization on prescribed sleep medications for ≥6 months. Ineligibility criteria included presence of obstructive sleep apnea or periodic limb movement disorder via ambulatory polysomnography. Fully eligible participants were randomized to either the psychosocial intervention or to a control condition providing psychoeducation on living well with cancer, blinded to condition. Assessment procedures were repeated immediately following intervention and 6–8 weeks later. Participants were compensated $150 for postsurgical, postintervention, and follow-up assessments, each, and $10 for each intervention session.

#### Measures

##### Predictors

***Sociodemographic assessment*.** Age, race, and ethnicity were collected via self-report. Age was dichotomized such that individuals 18–59 years of age were categorized as younger adults, and individuals 60–89 years of age were categorized as older adults [22]. Participants identified race and ethnicity using categories delineated by the US Census Bureau [23]. Race was coded as non-White versus White; ethnicity was coded as Hispanic versus non-Hispanic; and minority racial/ethnic status was coded as non-White and/or Hispanic versus non-Hispanic White. Education level, employment status, and income were collected from the MacArthur Sociodemographic Questionnaire [24]. A composite SES score was derived from these three variables [25]. For the attrition sample of enrolled participants only, resident addresses were available. Utilizing ZIP codes and full addresses as needed, participant county of residence was identified. Counties of residence were first categorized according to the 2013 National Center for Health Statistics Urban-Rural Classification Scheme for Counties [26] and subsequently dichotomized as large metropolitan area (“1”) versus all other, more rural categories (“0”).

***Baseline intervention targets*.** Depressive symptomatology was assessed with the Beck Depression Inventory – Second Edition (BDI-II), a validated measure of cognitive and somatic symptoms of depression used in the general and cancer population [27]. Anxious symptomatology was assessed with the State-Trait Anxiety Inventory (STAI), a reliable measure of anxious symptoms both during evaluation and general anxiety for nonmedical and medical populations [28,29]. Pain was measured via the McGill Pain Questionnaire (MPQ), a validated measure of pain intensity, quality, and duration using respondent identification of adjectives that most accurately describe their pain among the general and cancer populations [30-33]. The global pain rating index and its sensory subscale were used as primary pain measures [30]. The Pittsburgh Sleep Quality Index (PSQI), a validated measure of sleep quality within the past month, was used to measure sleep difficulties via the global score of the Sleep Quality Index [18,34,35].

##### Outcomes

***Outcome 1: Enrollment status among eligible patients*.** Approached eligible patients were classified into two groups: (1) Eligible Decliners (*N* = 181) and (2) Eligible Enrollees (*N* = 115). Excluding ineligible patients from Outcome 1 analyses, eligible enrollment status was dichotomized such that 0 = Eligible Decliner and 1 = Eligible Enrollee.

***Outcome 2: Eligibility status among all approached patients*.** All approached patients were classified into three groups: (1) Ineligible (*N* = 157); (2) Eligible Decliners (*N* = 181); and (3) Eligible Enrollees (*N* = 115). Eligibility status was dichotomized such that Ineligible patients (*N* = 157) were coded as “0” and Eligible patients, comprised of both Eligible Decliners (*N* = 181) Eligible Enrollees (*N* = 115), were coded as “1.”

***Outcome 3: Level of interest in study participation among nonenrollees*.** Patients who were either ineligible or declined participation indicated their interest level using a 3-point Likert scale (1 = Not at all interested; 2 = Somewhat interested; and 3 = Very interested. Trial 1 did not solicit reasons for decline among approached patients and did not contribute data to analyses evaluating decline motivation.

***Outcome 5: Study attrition*.** Trial 1 participant attrition was defined by study discontinuation prior to completing full procedures, due to voluntary patient withdrawal, loss to follow-up, or death. Participants who were discontinued from the study prematurely due to ineligibility documented after enrollment or who were withdrawn involuntarily by study staff were not considered to have experienced attrition.

### Trial 2: Yoga Intervention

#### Participants, recruitment, and procedures

Patients approached were women with confirmed gynecologic, gastrointestinal, or thoracic cancers for participation in a pilot feasibility study assessing the effects of a yoga program including mindfulness-based, relaxation, and gentle stretching techniques (NCT03385577; https://clinicaltrials.gov/ct2/show/NCT03385577), funded by the UF Health Cancer Center. Screening, recruitment, and informed consent procedures and associated training in patient-centered, culturally sensitive approaches identically modeled that of Trial 1 at the UF Health Gynecologic and Medical Oncology clinics. Inclusion criteria were (1) diagnosis with gynecologic, gastrointestinal, or thoracic cancer within the past year, (2) age of ≥18 years old, and (3) English fluency. Exclusionary criteria were (1) severe, uncontrolled psychopathology and/or suicidality, (2) bipolar disorder, (3) neurocognitive disorder, (4) psychosis; (5) participation in weekly yoga for 6 consecutive months within the past 5 years; (6) pregnancy; and (7) inability to independently fulfill basic needs.

Preliminarily eligible patients were approached for full screening and enrollment. Individuals who declined participation provided reason(s) for declined enrollment via standardized form. Enrollees were then added to a waitlist from which staff formed 2- to 6-person cohorts according to diagnostic site. At the start of their first yoga session at UF Health, participants completed a baseline assessment examining sociodemographic characteristics, mood, anxiety, sleep disturbance, and cancer-related distress. After weekly engagement in the 10-session program, assessment procedures were completed. Participants were compensated $10 per session.

#### Measures

##### Predictors

***Sociodemographic indicators*.** Age, race, and ethnicity among all approached patients and rurality status among Trial 2 participants were collected and codified using procedures identical to those described for Trial 1. Insurance status was utilized as the primary indicator for SES (“0” = uninsured, “1” = insured via private insurance or through CMS).

***Baseline intervention targets*.** Depressive symptoms, anxiety, and sleep disturbance among Trial 2 participants were collected utilizing identical measures to those described in Trial 1. Pain did not constitute a primary intervention target for Trial 2 and was thus neither measured not evaluated as a predictor of attrition.

##### Outcomes

***Outcome 1: Enrollment status among eligible participants*.** Yoga patients were classified into (1) Eligible Decliners (*N* = 144) and (2) Eligible Enrollees (*N* = 95).

***Outcome 4: Trends in reasons for study decline*.** Patients who declined enrollment in the yoga trial completed a form in which they endorsed or denied each of the following reasons for declining: inadequate time, overwhelm, distance, perceived poor health, lack of distress surrounding diagnosis, safety concerns, anticipating negative experiences with yoga, past negative experiences with yoga, religious reasons, refusal to respond, or other. Patients endorsed as many options as were relevant to their decline reason, then indicated their age range, race, and ethnicity. Separate analyses for each dichotomized decline reason (i.e., 0 = not endorsed, 1 = endorsed) were conducted with each aforementioned sociodemographic indicator.

***Outcome 5: Attrition*.** Trial 2 attrition was operationalized identically to that of Trial 1.

### Statistical Analysis

Descriptive statistics were used to examine enrollment and attrition rates. Aim 1 used chi-square analyses and accompanying logistic and multinomial regressions, with dichotomized age, race, ethnicity, and minority status as separate predictors.

For Aim 2, separate survival analyses with accompanying general discrete-time models for each trial utilizing person-period datasets and binary logistic regressions were performed. Assessment timepoints from Trial 1 were collapsed into four periods: recruitment; presurgical assessment; immediate postsurgical period and intervention; and postintervention measurement and 6- to 8-week follow-up. The four timepoints for Trial 2 attrition included enrollment through intervention commencement; sessions 1–3; sessions 4–7; and sessions 8–10. For each trial, unconditional growth and subsequent models separately incorporating each of the following predictors were conducted: age; race; ethnicity; minority status; composite SES score (Trial 1) and insurance status (Trial 2); and continuous baseline scores on psychosocial measures.

## Results

### Participant Characteristics

Six hundred ninety-two patients were approached for recruitment to Trial 1 and Trial 2 combined. Three hundred eighty-nine of these individuals across trials (56.2%) were 60 years of age or older. Among the full approached sample across trials, 15.9% were non-White and 5.1% were Hispanic, with 20.3% total identifying as a racial and/or ethnic minority among individuals for whom full data were available. Among the 539 approached for the Trial 1, 29.8% screened negative for insomnia and were ineligible for enrollment. Among the remaining total 535 eligible patients approached across both interventions, 60.7% declined enrollment and 39.3% completed the informed consent process. Thus, enrollees comprised 30.3% of all patients approached across Trial 1 and Trial 2.

### Combined Trial Analyses (Outcome 1)

#### Outcome 1: Enrollment status among eligible patients

No significant differences in decision to participate among eligible patients were observed comparing patients younger than 60 years old to patients 60 years and older; White to non-White patients; and non-Hispanic White patients to racial and/or ethnic minority patients combining Trial 1 and Trial 2 data. However, examining ethnicity, eligible Hispanic patients were 3.57 times more likely to enroll compared to their non-Hispanic counterparts (*p* = 0.020; Table 2). Further, patients 60 years of age and older were 3.82 times less likely to enroll compared to their younger counterparts, demonstrating a nonsignificant trend for lower likelihood of enrollment among eligible older adults (*p* = 0.087; Table 2).

**Table 2.** Chi-square analyses across trial accrual outcomes

Outcome 1: Enrollment status among eligible patients				Combined Trials 1 and 2			
		Declined n (%)	Enrolled n (%)	*χ*^2^		*p*	*ϕ*_*c*_
	Age (n = 535)			2.938		0.087	0.074
	Younger than 60 years old	141 (56.9%)	107 (43.1%)				
	60 years and older	184 (64.1%)	103 (35.9%)				
	Race (n = 528)			0.002		0.961	0.002
	White	266 (60.2%)	176 (39.8%)				
	Non-White	52 (60.5%)	34 (39.5%)				
	Ethnicity (n = 490)			**9.711**		**0.002**	**0.141**
	Non-Hispanic	**278 (60.0%)**	**185 (40.0%)**				
	Hispanic	**8 (29.6%)**	**19 (70.4%)**				
	Minority Status (n = 529)			2.578		0.108	0.070
	Non-Hispanic White	260 (62.1%)	159 (37.9%)				
	Racial/Ethnic minority	59 (53.6%)	51 (46.4%)				
Outcome 2: Eligibility status among all approached patients				Trial 1			
		Ineligible n (%)	Eligible n (%)	*χ*^2^		*p*	*ϕ*_*c*_
	Age (n = 453)			**5.910**		**0.015**	**0.144**
	Younger than 60 years old	**56 (29.2%)**	**136 (70.8%)**				
	60 Years and older	**105 (40.2%)**	**156 (59.8%)**				
	Race (n = 451)			0.020		0.887	0.007
	White	135 (35.3%)	247 (64.7%)				
	Non-White	25 (36.2%)	44 (63.8%)				
	Ethnicity (n = 441)			0.332		0.564	0.027
	Non-Hispanic	151 (35.4%)	276 (64.6%)				
	Hispanic	6 (42.9%)	8 (57.1%)				
	Minority Status (n = 452)			0.133		0.716	0.017
	Non-Hispanic White	130 (35.2%)	239 (64.8%)				
	Racial and/or Ethnic minority	31 (37.3%)	52 (62.7%)				
Outcome 3: Level of interest in study participation among nonenrollees				Trial 1			
		Not at all n (%)	Somewhat n (%)	Very n (%)	*χ*^2^	*p*	*ϕ*_*c*_
	Age (n = 326)				2.158	0.340	0.081
	Younger than 60 years old	20 (15.7%)	90 (70.9%)	17 (13.4%)			
	60 Years and older	40 (20.1%)	141 (70.9%)	18 (9.0%)			
	Race (n = 324)				3.083	0.214	0.098
	White	47 (17.0%)	200 (72.2%)	30 (10.8%)			
	Non-White	13 (27.7%)	30 (63.8%)	4 (8.5%)			
	Ethnicity (n = 321)				2.300	0.317	0.085
	Non-Hispanic	58 (18.5%)	223 (71.0%)	33 (10.5%)			
	Hispanic	1 (14.3%)	1 (57.1%)	28.6%			
	Minority status (n = 325)				2.519	0.284	0.088
	Non-Hispanic White	46 (17.0%)	196 (72.3%)	29 (10.7%)			
	Racial and/or ethnic minority	14 (25.9%)	34 (63.0%)	6 (11.1%)			
Outcome 4: Trends in reasons for study decline				Trial 2			
	Inadequate time	Not endorsed n (%)	Endorsed n (%)	*χ*^2^		*p*	*ϕ*_*c*_
	Age			3.102		0.078	0.147
	Younger than 60 years old	42 (65.6%)	22 (34.4%)				
	60 years and older	63 (78.8%)	17 (21.3%)				
	Race			3.730		0.053	0.164
	White	85 (75.2%)	28 (24.8%)				
	Non-White	14 (56.0%)	11 (44.0%)				
	Ethnicity			0.001		0.974	0.003
	Non-Hispanic	72 (72.0%)	28 (28.0%)				
	Hispanic	5 (71.4%)	2 (28.6%)				
	Minority status			3.807		0.051	0.165
	Non-Hispanic White	82 (75.9%)	26 (24.1%)				
	Racial and/or Ethnic minority	18 (58.1%)	13 (41.9%)				
	Stress and Overwhelm	Not endorsed n (%)	Endorsed n (%)	*χ*^2^		*p* or FET*	*ϕ*_*c*_
	Age			3.203		0.074	0.149
	Younger than 60 years old	58 (90.6%)	6 (9.4%)				
	60 Years and older	78 (97.3%)	2 (2.5%)				
	Race			0.181		0.671	0.036
	White	106 (93.8%)	7 (6.2%)				
	Non-White	24 (96.0%)	1 (1.0%)				
	Ethnicity			4.818		0.085	0.212
	Non-Hispanic	94 (94.0%)	6 (6.0%)				
	Hispanic	5 (71.4%)	2 (28.6%)				
	Minority Status			1.131		0.377*	0.090
	Non-Hispanic White	103 (95.4%)	5 (4.6%)				
	Racial and/or Ethnic minority	28 (90.3%)	3 (9.7%)				
	Distance	Not endorsed n (%)	Endorsed n (%)	*χ*^2^		*p*	*ϕ*_*c*_
	Age			0.216		0.642	0.039
	Younger than 60 years old	14 (21.9%)	50 (78.1%)				
	60 Years and older	15 (18.8%)	65 (81.3%)				
	Race			**10.612**		**0.001**	**0.277**
	White	17 (15.0%)	96 (85.0%)				
	Non-White	11 (44.0%)	14 (56.0%)				
	Ethnicity			0.181		1.000	0.041
	Non-Hispanic	21 (21.0%)	79 (79.0%)				
	Hispanic	1 (14.3%)	6 (85.7%)				
	Minority status			**7.696**		**0.006**	**0.235**
	Non-Hispanic White	17 (15.7%)	91 (84.3%)				
	Racial and/or Ethnic minority	12 (38.7%)	19 (61.3%)				
	Poor health	Not endorsed n (%)	Endorsed n (%)	*χ*^2^		*p* or FET*	*ϕ*_*c*_
	Age			3.327		0.068	0.152
	Younger than 60 years old	61 (95.3%)	3 (4.7%)				
	60 Years and older	69 (86.3%)	11 (13.8)				
	Race			0.238		0.626	0.042
	White	103 (91.2%)	10 (8.8%)				
	Non-White	22 (88.0%)	3 (12.0%)				
	Ethnicity			0.130		0.543	0.035
	Non-Hispanic	90 (90.0%)	10 (10.0%)				
	Hispanic	6 (85.7%)	1 (14.3%)				
	Minority status			0.593		0.436*	0.065
	Non-Hispanic White	99 (91.7%)	9 (8.3%)				
	Racial and/or Ethnic minority	27 (87.1%)	4 (12.9%)				
	No perceived distress	Not endorsed n (%)	Endorsed n (%)	*χ*^2^		*p* or FET*	*ϕ*_*c*_
	Age			0.839		0.360	0.076
	Younger than 60 years old	57 (89.1%)	13 (16.3%)				
	60 Years and older	67 (83.8%)	7 (10.9%)				
	Race			0.056		0.813	0.020
	White	97 (85.8%)	16 (14.2%)				
	Non-White	21 (84.0%)	4 (16.0%)				
	Ethnicity			1.316		0.254	0.111
	Non-Hispanic	87 (87.0%)	13 (13.0%)				
	Hispanic	5 (71.4%)	2 (28.6%)				
	Minority Status			0.799		0.390*	0.076
	Non-Hispanic White	94 (87.0%)	14 (13.0%)				
	Racial and/or Ethnic minority	25 (80.6%)	6 (19.4%)				
	Past yoga experience	Not endorsed n (%)	Endorsed n (%)	*χ*^2^		*p* or FET*	*ϕ*_*c*_
	Age			1.252		0.263	0.093
	Younger than 60 years old	60 (93.8%)	4 (6.3%)				
	60 Years and older	78 (97.5%)	2 (2.5%)				
	Race			1.148		0.284	0.091
	White	108 (95.6%)	5 (4.4%)				
	Non-White	25 (100%)	0 (0.0%)				
	Ethnicity			1.066		0.340	0.100
	Non-Hispanic	95 (95.0%)	5 (5.0%)				
	Hispanic	6 (85.7%)	1 (14.3%)				
	Minority status			0.016		1.000*	0.011
	Non-Hispanic White	104 (96.3%)	4 (3.7%)				
	Racial and/or Ethnic minority	30 (96.8%)	1 (3.2%)				
	Aversion to yoga	Not endorsed n (%)	Endorsed n (%)	*χ*^2^		*p* or FET*	*ϕ*_*c*_
	Age			0.000		1.000	0.000
	Younger than 60 years old	56 (87.5%)	8 (12.5%)				
	60 Years and older	70 (87.5%)	10 (12.5%)				
	Race			0.383		0.536	0.053
	White	100 (88.5%)	13 (11.5%)				
	Non-White	21 (84.0%)	4 (16.0%)				
	Ethnicity			2.266		0.176	0.146
	Non-Hispanic	90 (90.0%)	10 (10.0%)				
	Hispanic	5 (71.4%)	2 (28.6%)				
	Minority status			1.887		0.212*	0.117
	Non-Hispanic White	97 (89.8%)	11 (10.2%)				
	Racial and/or Ethnic minority	25 (80.6%)	6 (19.4%)				
	Safety concerns	Not endorsed n (%)	Endorsed n (%)	*χ*^2^		*p* or FET*	*ϕ*_*c*_
	Age			0.025		0.873	0.013
	Younger than 60 years old	63 (98.4%)	1 (1.6%)				
	60 Years and older	79 (98.8%)	1 (1.3%)				
	Race			1.391		0.238	0.100
	White	112 (99.1%)	1 (0.9%)				
	Non-White	24 (96.0%)	1 (4.0%)				
	Ethnicity			0.071		1.000*	0.026
	Non-Hispanic	99 (99.0%)	1 (1.0%)				
	Hispanic	7 (100.0%)	0 (0.0%)				
	Minority status			0.898		0.398*	0.080
	Non-Hispanic White	107 (99.1%)	1 (0.9%)				
	Racial and/or Ethnic minority	30 (96.8%)	1 (3.2%)				
	Religious reasons	Not endorsed n (%)	Endorsed n (%)	*χ*^2^		*p*	*ϕ*_*c*_
	Age			1.623		0.203	0.106
	Younger than 60 years old	64 (100.0%)	0 (0%)				
	60 Years and older	78 (97.5%)	2 (2.5%)				
	Race			0.223		0.637	0.040
	White	112 (99.1%)	1 (0.9%)				
	Non-White	25 (100.0%)	0 (0.0%)				
	Ethnicity			6.296		0.127	0.243
	Non-Hispanic	99 (99.0%)	1 (1.0%)				
	Hispanic	6 (85.7%)	1 (14.3%)				
	Minority status			0.898		0.398	0.080
	Non-Hispanic White	107 (99.1%)	1 (0.9%)				
	Racial and/or Ethnic minority	30 (96.8%)	1 (3.2%)				
	Other	Not endorsed n (%)	Endorsed n (%)	*χ*^2^		*p* or FET*	*ϕ*_*c*_
	Age			2.618		0.106	0.135
	Younger than 60 years old	56 (87.5%)	8 (12.5%)				
	60 Years and older	76 (95.0%)	4 (5.0%)				
	Race			**4.914**		**0.027**	**0.189**
	White	106 (93.8%)	7 (6.2%)				
	Non-White	20 (80.0%)	5 (20.0%)				
	Ethnicity			0.688		1.000	0.080
	Non-Hispanic	91 (91.0%)	9 (9.0%)				
	Hispanic	7 (100.0%)	0 (0.0%)				
	Minority status			2.842		0.139*	0.143
	Non-Hispanic White	101 (93.5%)	7 (6.5%)				
	Racial and/or Ethnic minority	83.9%	5 (16.1%)				

Boldface values = statistical significance (*p* < .05).

*FET = Fisher’s exact test applied where cells generate expected counts <5.

### Trial 1 Analyses (Outcomes 2–3, 5)

#### Outcome 2: Eligibility status among all approached patients

Patients 60 years of age or older were significantly less likely to be eligible for enrollment compared to their younger counterparts (*p* = 0.015) in Trial 1, such that the odds of eligibility for a patient younger than 60 years of age were 1.60 times that of an older patient (Table 2). Results indicated no significant differences in eligibility based on dichotomized race, ethnicity, or minority status in Trial 1 (Table 2).

#### Outcome 3: Level of interest in study participation among nonenrollees

Based on available data from Trial 1, no significant differences in participation interest based on dichotomized age, race, ethnicity, or minority status were observed (Table 2).

#### Outcome 5: Study attrition

Fifty-one (44%) of the 115 Trial 1 enrollees were excluded for one of the following reasons post-surgery: a lack of an inclusionary gynecologic cancer, a diagnosis of obstructive sleep apnea, or a lack of clinical insomnia. Thus, 56% of enrollees were fully eligible for randomization at post-surgery or were eligible at the point in which they (a) withdrew from the study, (b) were withdrawn by the PI, or (c) were lost to follow-up. These 64 participants comprised the attrition analyses sample and were predominantly White (*N* = 54, 84.4%), non-Hispanic (*N* = 60, 96.8%), and non-Hispanic White (*N* = 52, 81.3%), demonstrating similar proportions to those of the full approached sample for this trial.

A survival analysis using a person-period dataset was conducted to determine overall Trial 1 attrition rate. The resulting life table reflected a cumulative proportional survival rate of 59%, indicating the portion of participants remaining in the protocol by the end of the study period. Contrary to hypotheses, results reflected no significant differences in likelihood of attrition based on age, race, ethnicity, minority status, SES, rurality status, group assignment, or diagnostic site). Similarly, no significant differences in likelihood of attrition based on BDI-II, STAI, MPQ, and PSQI scores were observed (Table 3).

**Table 3.** Predictors of attrition in Trial 1 (CBTi.p trial) (outcome 5) (*N* = 64)

	*B*	*df*	*p*	Exp(*B*)
Age	−0.088	1	0.825	0.916
Race	0.583	1	0.275	1.792
Ethnicity	−19.583	1	0.999	0.000
Minority status	0.150	1	0.771	1.162
SES	0.005	1	0.975	1.005
Large metropolitan area	−19.683	1	0.999	0.000
	*B*	*df*	*p*	Exp(*B*)
BDI	−0.025	1	0.602	0.975
STAI				
State	0.007	1	0.779	1.007
Trait	−0.015	1	0.600	0.985
MPQ				
Total	0.001	1	0.957	1.001
Sensory subscale	0.014	1	0.600	1.014
PSQI	0.005	1	0.924	1.005

BDI, Beck Depression Inventory – Second Edition; MPQ, McGill Pain Questionnaire; PSQI, Pittsburgh Sleep Quality Index; SES, socioeconomic status; STAI, State-Trait Anxiety Inventory.

### Trial 2 Analyses (Outcomes 4–5)

#### Outcome 4: Trends in reasons for study decline

Examining reasons for decline among Trial 2 participants, non-Hispanic White participants were significantly more likely to endorse decline due to distance from the research site (*p* < 0.01; Table 2). Further, younger (*p* = 0.078) and racial and/or ethnic minority patients (*p* = 0.051) demonstrated nonsignificant trends toward higher likelihood of endorsing decline due to inadequate time to participate. Hispanic patients demonstrated a nonsignificant trend toward higher likelihood of endorsing decline due to feeling overwhelmed relative to their non-Hispanic counterparts (*FET* = 0.085). Older patients nonsignificantly trended toward higher likelihood of endorsing perceived inadequate health status as a reason for decline in comparison to those approached younger than 60 years old (*p* = 0.068).

#### Outcome 5: Study attrition

At the time of analysis, 95 participants were enrolled in Trial 2 and reflected a predominantly White (85.3%), non-Hispanic (88.4%), and non-Hispanic White (75.8%) sample. A survival analysis assessing Trial 2 attrition reflected a cumulative proportional survival rate of 66%, indicating the portion of participants remaining in the protocol by the end of the study period. As with Trial 1, no significant differences in likelihood of attrition based on age, race, ethnicity, minority status, insurance status, and diagnostic group were observed in Trial 2. Similarly, no significant differences in likelihood of attrition based on BDI-II, STAI, and PSQI scores were observed (Table 4). Regarding rurality, however, participants residing in large fringe or central metropolitan areas were 5.02 times more likely to drop out of Trial 2 than their more rural counterparts (*B* = 1.613, *p* = 0.026), subverting hypotheses and diverging from attrition results observed in Trial 1.

**Table 4.** Predictors of attrition in Trial 2 (yoga trial) (outcome 5) (*N* = 95)

	*B*	*df*	*p*	Exp(*B*)
Age	−0.160	1	0.652	1.173
Race	0.511	1	0.294	1.668
Ethnicity	0.212	1	0.696	1.236
Minority status	0.535	1	0.190	1.708
Insurance coverage	0.038	1	0.947	1.038
Large metropolitan area	1.613	1	**0.026**	5.020
	*B*	*df*	*p*	Exp(*B*)
BDI	−0.061	1	0.186	0.941
STAI				
State	0.043	1	0.112	1.044
Trait	0.014	1	0.660	1.014
PSQI	0.043	2	0.694	1.044

BDI, Beck Depression Inventory; PSQI, Pittsburgh Sleep Quality Index; STAI, State-Trait Anxiety Inventory.

Boldface values = statistical significance (*p* < .05).

## Discussion

Our results reflect significant barriers to optimizing accrual and retention rates in both behavioral CCTs examined, as is consistent with extant evidence revealing CCT participation patterns [2]. The overall enrollment rate among all approached eligible participants across trials was 39.3%. Among eligible enrollees prior to the start of Trial 1 and Trial 2, total attrition rates were 50.0% and 56.8%, respectively. While recruitment data specific to *behavioral* trials are limited, the present study demonstrates comparable outcomes to existing studies, with enrollment rates of previous behavioral CCTs ranging from 25.6% to 59.4% [17,36].

Older adults were less likely to fulfill eligibility criteria in Trial 1 and trended toward lower likelihood of enrolling even if eligible. Considering general and cancer-related evidence on older adult health, these approached patients were likely more susceptible to exclusionary criteria regarding functional status [37] and neurocognitive decline [38], and as indicated in decline reason trends in Trial 2 and global enrollment decision trends, may have perceived themselves as having inadequate functional status for enrollment. Further, given the known disparities in sleep disorders that disproportionately affect older adults [39], it is possible that older patients were more likely ineligible due to disqualifying sleep-related comorbidities in Trial 1. Further, in spite of normative changes resulting in lighter sleep, less total sleep time, and more nighttime awakenings in comparison to younger adults, older patients approached for Trial 1 were less likely to achieve Pittsburgh Sleep Quality Index scores greater than 5 or screen eligible via insomnia screen (*p* = 0.021), as is consistent with limited perceptions and/or underreporting of sleep concerns common in this population [40].

Our findings indicate no significant differences in enrollment decision among eligible patients across age, race, or minority status, and even reflect higher likelihood of enrollment among Hispanic approached patients. This diverges from the amalgam of extant evidence, which dependably demonstrates minority CCT underrepresentation even in behavioral CCTs [3-5]. However, as is consistent with the existing literature [3,9], our findings indicate no sociodemographic differences in participation interest level among patients who screened ineligible or who declined participation. As such, these results – that is, demonstrating lack of sociodemographic differences in interest level among nonenrollees – support that enduring underrepresentation of minority patients in CCTs may reside less in a comparative lack of interest and more in persisting barriers to CCT awareness, opportunities, and enrollment; differences in perceptions regarding CCT participation despite similar interest level; and other potentially unknown factors that disproportionately restrict their participation in CCTs relative to non-Hispanic White patients. Nonetheless, closure of the minority gap and increased diversity in CCT participation is critical, especially in the context of disparities in treatment quality and survival that negatively impact minority patients [41].

Interestingly, chi-square analyses examining the relationships between minority status and endorsement of various decline motivations demonstrated higher likelihood among non-Hispanic White decliners to report distance as a primary reason for decline in Trial 2 than their racial and/or ethnic minority counterparts. Given the inextricable relationships between race, ethnicity, and SES, these results might be explained by racialized differences in SES and access to transportation to receive care at UF Health among geographically distant minority patients relative to their non-Hispanic White counterparts. Further, provided with the aforementioned trends toward minority patients reporting inadequate time more often than their non-Hispanic White counterparts, these results collectively accentuate the impetus for further investigation of sociodemographic differences in decision-making related to minority enrollment in CCTs.

Regarding attrition in both trials, no significant differences were observed across most sociodemographic predictors, contrasting with our hypothesis derived from what has typically been observed in both general and CCT retention. Further subverting expectations, urban participants originating from large metropolitan areas were more likely to drop out of the yoga intervention than their more rural counterparts, despite the authors hypothesizing significance in the opposite direction due to extant literature suggesting elevated barriers to CCT participation among patients of rural residence [42,43]. Based off the origin cities of participants enrolled yoga intervention sample, these findings may be best understood in the context of greater physical distance from the study site rather than the effects of urbanity *per se*. For example, in comparison to more rural participants living closer to the medium metropolitan county of Alachua – that is, where intervention sessions occurred – participants from large metropolitan areas traveled from counties much further from the study site, such as Duval, Lake, and Clay County. These same differences were likely not observed in the CBTi.p. intervention due to occurrence of sessions within the homes of participants. Regarding other predictors of attrition assessed, no differences were observed in attrition rate based on baseline levels of mood, anxiety, pain, sleep disturbance, or diagnostic site characteristics. It is possible that these results reflect a lack of differences in perceived benefit of sustained participation in the study regardless of severity of problems related to mood, anxiety, pain, or sleep, and that level of symptomatology was not prohibitive in maintaining enrollment.

These analyses contribute to an improved understanding of the complex sociodemographic recruitment trends observed regarding representation of the underserved in behavioral CCTs. Specifically, this study offers a novel contribution in its use of survival analyses and accompanying general discrete-time models to determine potential sociodemographic differences in attrition throughout participation. Given subversion of typical minority CCT accrual trends in (1) higher relative Hispanic enrollment and (2) lack of accrual disparities disproportionately impacting approached minority patients, these results may contribute to a preliminary groundwork for developing best practices for minority CCT recruitment and retention. In both parent trials, cultural sensitivity was of the utmost importance, prioritizing several patient-centered strategies to optimize diverse recruitment in their (1) equal-opportunity health record review, (2) strong relationships between research staff and the physicians, nurses, and clinical support, (3) enthusiastic physician involvement, (4) diverse representation among research and clinical staff, (5) commitment to tailored psychoeducation, and (6) marked persistence in spite of confrontation with systematic clinical obstacles commonly cited in the literature [10].

While commitment to these strategies was ineffective in optimizing *absolute* minority recruitment in the context of low overall accrual rates across approached samples, these strategies may have served an important role in subverting the typical pattern of *relative* minority underrepresentation among patients approached at clinical recruitment sites, in that not only were eligible minority patients not less likely to enroll but also in that eligible Hispanic patients among them were more likely to enroll than their non-Hispanic counterparts. These techniques may thus yield important implications for optimizing relative research participation among underserved populations in cancer; however, further investigation is necessary for expanding the implications of relative improvements in dismantling barriers to maximizing absolute accrual rates across sociodemographic characteristics. Accompanied by such research and resulting improvements in overall accrual rates, these patient-centered, culturally sensitive strategies that have positively impacted relative enrollment of minority patients may wield larger, downstream implications for care enhancement and overall attenuation of cancer outcome disparities among marginalized patients.

### Study Limitations

Nonetheless, the present study had several limitations. The overarching limitation of these analyses resides in overall low accrual and retention and limited diversity of the studies from which they are derived, resulting in decreased statistical power and generalizability of findings. Further, this limitation necessitated sociodemographic dichotomization, obscuring potential nuance in outcomes across target underrepresented groups in both trials. It is likely that this limited diversity is attributable to internal recruitment methods without supplemental community-based strategies, as use of clinical infrastructure alone inherently circumnavigates patients who cannot access the facilities from which participants are recruited, among whom minority patients are overrepresented [5,8]. In addition, while higher enrollment was observed among approached eligible Hispanic patients, significant ethnic differences observed in enrollment are qualified by the exclusion of non-English-speaking patients. Therefore, it is possible that with the inclusion of monolingual, Spanish-speaking Hispanic participants, the observed higher likelihood of enrollment among Hispanic patients relative to their non-Hispanic counterparts would have been attenuated, insignificant, or even reversed. Future intervention studies should incorporate multilingual methods such that the effects of linguistic diversity can be accounted for in optimizing recruitment methods.

### Clinical Implications

These results provide a strong rationale for further implementing patient-centered, culturally sensitive accrual and attrition prevention strategies to minimize relative disparities in recruitment outcomes, while emphasizing necessity for increased focus on optimizing diversity in full approached samples and absolute accrual rates across sociodemographic characteristics. The recruitment techniques employed in the parent interventions may contribute to a framework of optimal practices for enrolling and retaining minority cancer patients for future CCTs from a relative perspective, wherein these studies exhibited lack of both enrollment, interest, and retention disparities, and even greater likelihood of enrollment among Hispanic patients [5-7,44]. Taken together, the limited diversity characterizing the patient samples approached for parent CCT participation, overall accrual rates across sociodemographic characteristics observed in each trial, and both absolute attrition and relative retention disparities revealed suggests that future CCT recruitment among minority and urban populations might benefit from community-based strategies that supplement internal recruitment through cancer care facilities. Comprehensive approaches will be essential to improving access for individuals with cancer who are underserved in formal cancer care and will provide better avenues for optimizing opportunities among underserved patients to participate in CCTs. The aims of future research in CCT recruitment should also accentuate techniques for optimizing age inclusivity when developing eligibility criteria, creatively accounting for confounding factors to which older cancer patients are more susceptible and retaining sufficiently rigid protocols to maximize both internal and external validity.

Our findings in retention in the yoga trial suggest need for further investigation into resolution of distance-related barriers to sustained CCT participation. These results are of particular importance, given the enduring impacts of the COVID-19 pandemic in encouraging increased adoption of remote intervention administration methods to optimize participant safety and public health. While the data analyzed from both target behavioral CCTs emerged entirely from cohorts utilizing direct, in-home, or on-site intervention methods, the yoga trial was resumed following COVID-19 transitioned to a virtual administration platform. Given that participants located in large metropolitan areas, frequently further away from the trial location site, were significantly more likely to discontinue yoga study participation prior to completion, it is feasible to expect that virtual administration may attenuate these disparities in attrition by affording participants the ability to participate in the intervention without the addition of travel burden. By contrast, due to potential technological limitations and associated geographical disparities in Internet access, virtual administration may reverse these inequities in retention and compromise participation among rural participants with greater obstacles to participation via Internet-reliant methods. While accrual and retention data remain limited on virtual administration of the yoga intervention, it will be critical to examine how remote administration may mitigate, magnify, or reverse the geographical attrition disparities observed in its live administration.

According to the results of its live administration as analyzed, the implications of the present study support further training and development in CCT recruitment approaches; investigation into culturally tailored methods for diverse trial enrollment and attrition prevention; and diversification of recruitment sites to improve access to CCTs among socioeconomically disenfranchised individuals with cancer, and thereby optimize absolute accrual rates and sample diversity in applying techniques conducive to representative sampling among patients approached for CCTs. Finally, while addressing these barriers that persist at the microcosm of individual protocols, it is imperative that investigations prioritizing diverse CCT recruitment are contextualized within the systemic, societal barriers that inform sociodemographic disparities in CCT recruitment and cancer-related outcomes.
